# Novel Dolphin Tupavirus from Stranded Atlantic White-Sided Dolphin with Severe Encephalitis, Canada, 2024

**DOI:** 10.3201/eid3111.251203

**Published:** 2025-11

**Authors:** Oksana Vernygora, Laura Bourque, Megan Jones, Ole Nielsen, Carissa Embury-Hyatt, Estella Moffat, Tonya Wimmer, Oliver Lung

**Affiliations:** National Centre for Foreign Animal Disease, Winnipeg, Manitoba, Canada (O. Vernygora, C. Embury-Hyatt, E. Moffat, O. Lung); Canadian Wildlife Health Cooperative, Atlantic Region, Charlottetown, Prince Edward Island, Canada (L. Bourque, M. Jones); Department of Fisheries and Oceans Canada, Winnipeg (O. Nielsen); Marine Animal Response Society, Halifax, Nova Scotia, Canada (T. Wimmer); University of Manitoba, Winnipeg (O. Lung)

**Keywords:** tupavirus, viruses, rhabdovirus, Rhabdoviridae, dolphin, meningitis/encephalitis, cetaceans, Canada

## Abstract

We sequenced a novel rhabdovirus, *Tupavirus delphini* (dolphin tupavirus), from the brain of a stranded dead Atlantic white-sided dolphin with severe encephalitis in Canada. In situ hybridization linked presence of the virus to the animal’s brain pathology and death. Our findings underscore the importance of monitoring marine mammals for unexpected pathogens.

Cetaceans (whales and dolphins) are ubiquitous in the world’s oceans and are critical for monitoring oceanic ecosystem health (*1*). Despite their importance, little is known about diseases that affect free-ranging cetacean populations. Monitoring cetacean populations is challenging; consequently, much can be gained from necropsy examinations of dead, stranded animals. This study describes the discovery of a novel rhabdovirus species detected during the necropsy of an Atlantic white-sided dolphin (*Leucopleurus acutus*) found stranded on the Atlantic coast of Nova Scotia, Canada (Appendix 1 Figure 1).

Rhabdoviruses are a diverse group of enveloped single-stranded negative-sense RNA viruses that infect vertebrates, invertebrates, and plants. Most aquatic rhabdoviruses are described from ray-finned fish and amphibians; little is known about rhabdoviruses infecting marine mammals (*2*). Two species of rhabdoviruses are reported from cetaceans, dolphin rhabdovirus (*3*,*4*) and harbor porpoise rhabdovirus, neither of which was definitively linked to causing disease in their hosts (*5*). We characterized a novel rhabdovirus genome and used in situ hybridization to link viral infection with histopathologic lesions within the brain.

## The Study

**Figure 1:**
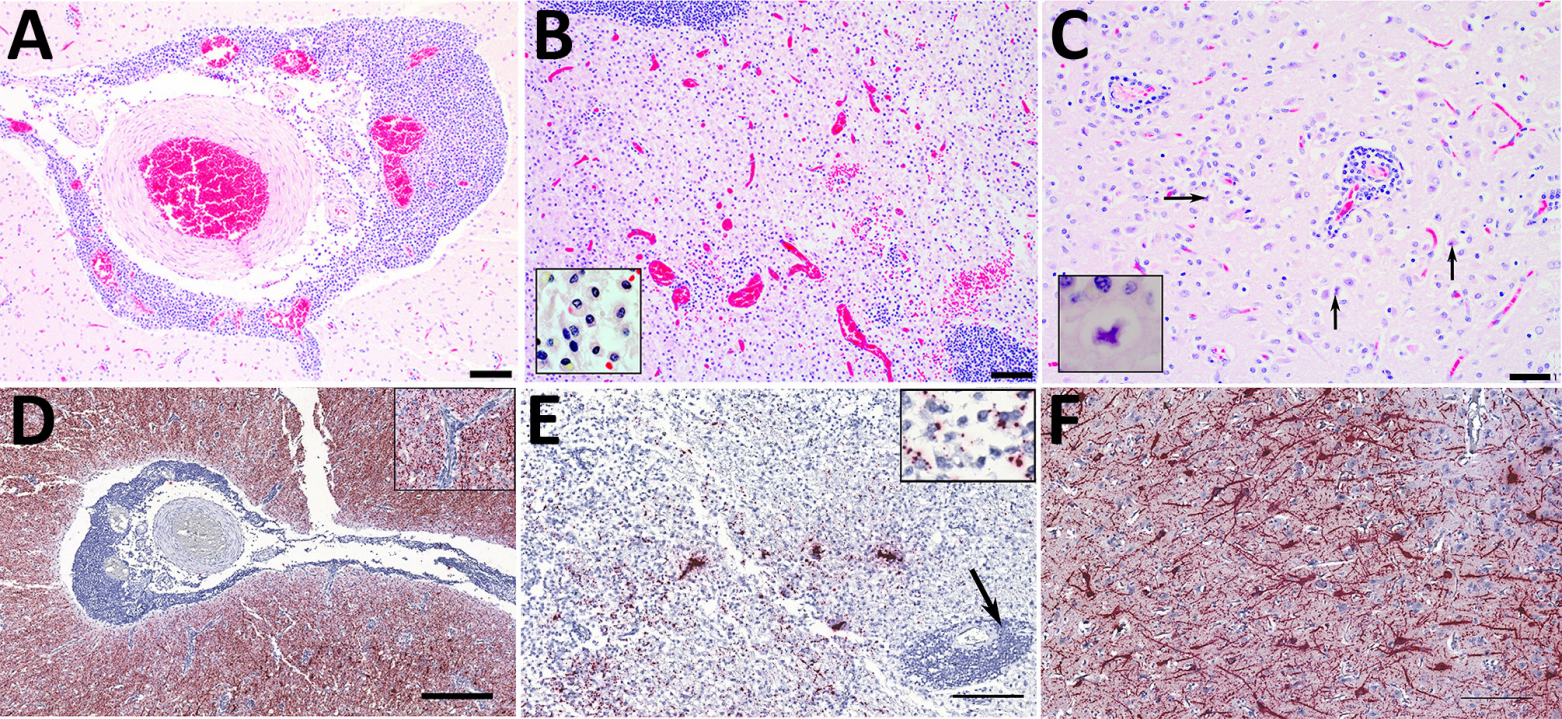
Histopathology (A–C) and in situ hybridization (D–F) of brain tissue from stranded Atlantic white-sided dolphin infected with novel tupavirus, Canada. Histopathology images are stained with hematoxylin and eosin; cytoplasm and connective tissues appear pink, red blood cells red, and cell nuclei purple. A) Cross-section through an arteriole within the neuropil at the level of the internal capsule, depicting sometimes massive infiltration of perivascular (Virchow-Robin) space by large numbers of lymphocytes and plasma cells, fewer numbers of macrophages, and rare eosinophils. Scale bar indicates 100 μm. B) Cross-section through the cingulate gyrus at the level of the internal capsule, showing almost complete effacement of normal neuropil architecture by massive numbers of hypertrophied microglial cells. Also depicted are multiple arterioles with large perivascular cuffs of lymphocytes and plasma cells, as well as multiple areas of hemorrhage within the neuropil. Inset shows microglial cells. Scale bar indicates 100 μm. C) Section through neocortex exhibiting extensive infiltration of the neuropil by glial cells, which frequently form nodules surrounding necrotic neurons. Necrotic neurons are shrunken with hypereosinophilic cytoplasm and condensed, pyknotic nuclei (arrows). There are also multiple small arterioles with lymphoplasmacytic perivascular cuffs. Inset shows a necrotic neuron. Scale bar indicates 50 μm. D) Abundant viral RNA (red staining) in the area surrounding the affected arteriole. Staining is observed in the neuropil as well as some glial cells; however, endothelial cells are not infected (inset). No staining is observed within the perivascular inflammatory cells. Scale bar indicates 400 μm. E) Viral RNA in microglial cells and neuropil (inset) but not within perivascular cuffs (arrow). Scale bar indicates 200 μm.) Abundant viral RNA within neurons and dendrites. In situ hybridization, paraffin-embedded formalin-fixed tissue sections were performed with RNAScope 2.5HD Detection Reagent and custom probes targeting the 1275–2283 nt region of the viral genome (Bio-Techne Advanced Cell Diagnostics, https://www.bio-techne.com). Scale bar indicates 200 μm.

On October 27, 2024, a freshly dead Atlantic white-sided dolphin was found ashore on La Bloc Beach in the Cape Breton Highlands National Park, Nova Scotia, Canada. The carcass was well preserved with minimal scavenging or postmortem decomposition (Appendix 1 Figure 1) and demonstrated overall good nutritional condition (*6*). No significant lesions were identified from the carcass other than those from severe encephalitis. We conducted diagnostic PCR testing of frozen brain tissue for common pathogens known to cause encephalitis in marine mammals (avian influenza virus, cetacean morbillivirus, herpesvirus, *Brucella ceti*, *Sarcocystis* sp., and *Toxoplasma gondii*); results were negative.

Examination of transverse sections of the brain revealed multiple extensive areas of severe inflammation and necrosis primarily throughout the forebrain within the regions of the cingulate gyrus, internal capsule, thalamus, and temporal and parietal lobes of the neocortex. In situ hybridization showed consistent presence of viral RNA in the lesions (Figure 1). Regions of the brain that did not exhibit lesions were primarily within the midbrain and hindbrain. In affected regions, we observed generalized mild to severe expansion of the perivascular space by lymphocytes and plasma cells and fewer macrophages and rare eosinophils (Figure 1, panel A). We observed intense positive staining for viral RNA in the surrounding neuropil but not in the perivascular inflammatory cells (Figure 1, panel D). In some areas, the normal tissue architecture was effaced by massive infiltration of hypertrophied microglial cells (Figure 1, panel B), many of which stained positive for viral RNA (Figure 1, panel E). In the neocortex, we noted glial nodules surrounding necrotic neurons (Figure 1, panel C), as well as neuronal cell bodies and dendrites within lesion areas that contained abundant viral RNA (Figure 1, panel F). All other tissues examined microscopically were either within normal limits or exhibited incidental lesions unrelated to the neuropathology described.

**Figure 2:**
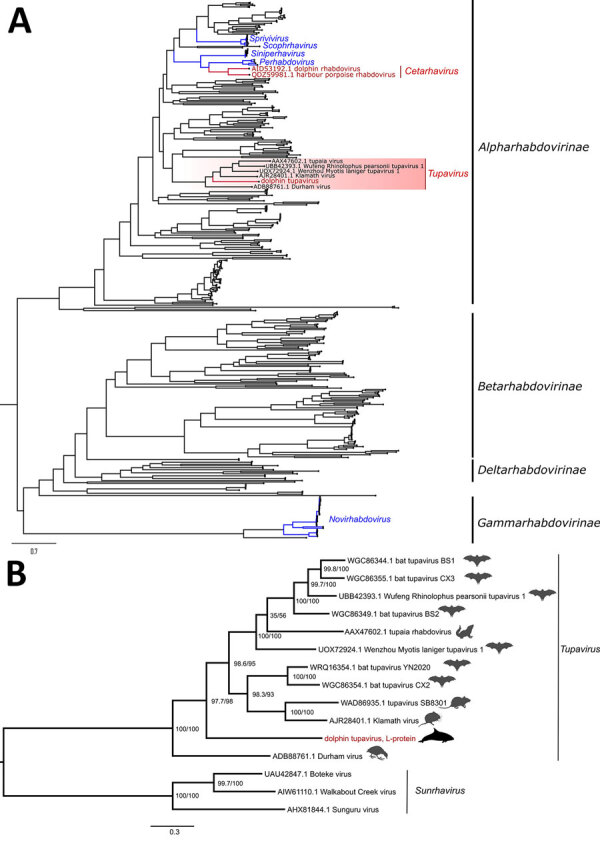
Maximum-likelihood phylogenetic trees of novel dolphin tupavirus from stranded Atlantic white-sided dolphin, Canada, and reference rhabdoviruses. Trees were reconstructed based on the full-length amino acid sequences of the polymerase protein. All sequence records for the phylogenetic analysis were downloaded from GenBank (Appendix 2 Table 2). Scale bars represent the estimated average number of substitutions per site. A) High-level phylogeny includes a subset of representative members across the *Rhabdoviridae* family (n = 438 sequence records) to determine the placement of the new virus within the family. Red text indicates rhabdoviruses of marine mammals, including the newly identified dolphin tupavirus; blue text indicates fish rhabdoviruses. The tree was rooted at the midpoint. B) Genus-level phylogeny provides a fine-level assessment of the phylogenetic affinities of the new virus within the *Tupavirus* genus. Red text indicates the sequence of the novel tupavirus; red shading highlights the *Tupavirus* clade in the phylogenetic tree. The phylogenetic bracket of the dolphin tupavirus includes a basal Durham virus and a large crown clade comprising the rest of the *Tupavirus* member viruses. Durham virus described from an American coot (*Fulica americana*) that demonstrated signs of severe disease associated with infection, including lesions isolated to the central nervous system and consisting of severe cerebral necrosis and mononuclear inflammation similar to DTV. Support values at the nodes were determined by SSH-aLRT/Ultrafast bootstrap. *Tupavirus* genus tree was rooted to the outgroup members of the *Sunrhavirus* genus, Boteke, Sunguru, and Walkabout Creek viruses.

For whole-genome sequencing, we performed sample preparation and data processing (*7*) with modifications (Appendix 1). The complete 11,088-nt dolphin tupavirus (DTV) genome we obtained (deposited into GenBank, accession no. PV683224) had a structure typical of rhabdoviruses comprising 5 major open reading frames (ORFs), nucleocapsid, phosphoprotein, matrix, glycoprotein, and RNA-dependent RNA polymerase (L) proteins, and a putative small hydrophobic protein between the matrix protein and glycoprotein, that encode proteins highly divergent from other rhabdoviruses (Table; Figure 2) (Appendix 1 Figure 2; Appendix 2 Table 1) (*8**,**9*). Phosphoprotein contains a putative C protein in an overlapping reading frame. Each ORF of the DTV genome is flanked by conserved transcription initiation (UUGUC) and termination/polyadenylation (AWCU7) signals and an inferred untranscribed intergenic sequence (GG or GA). The L protein of DTV contains an LDSPL motif, a modification of the animal rhabdovirus conserved motif (LNSPL), also found in Durham virus, another tupavirus (*10*,*11*).

**Table:** Characteristics of highly divergent novel dolphin tupavirus protein sequences from the brain of a stranded dead Atlantic white-sided dolphin with severe encephalitis, Canada*

	Nucleotide			Amino acid	
Protein	Top match by BLASTn	% Identity		Top match by DELTA-BLAST	% Identity
Nucleocapsid	Wufeng *Rhinolophus pearsonii* tupavirus 1 (GenBank accession no. MZ328291.1)	68.54		Wufeng bat tupavirus 2 (GenBank accession no. WPV62772.1)	54.88
Phosphoprotein	NA	NA		Wufeng *Rhinolophus pearsonii* tupavirus 1 (GenBank accession no. UBB42388.1)	17.55
Matrix protein	NA	NA		Bat tupavirus CX1 (GenBank accession no. WGC86350.1)	32.68
SH protein	NA	NA		Durham virus (GenBank accession no. ADB88762)	33.33
Glycoprotein	NA	NA		Wufeng bat tupavirus 2 (GenBank accession no. WPV62776.1)	30.62
Polymerase	Wenzhou *Myotis laniger* tupavirus 1 (GenBank accession no. OM030290.1)	55.42		Klamath virus (GenBank accession no. AJR28401.1)	55.36

*Percentage amino acid identity for the SH protein was calculated by aligning it to the SH from Durham virus, the closest tupavirus to DTV, using a BLASTp algorithm (https://blast.ncbi.nlm.nih.gov/Blast.cgi). SH proteins have very low identity among the members of the genus (*7**,**8*). NA, not applicable; SH, small hydrophobic protein.

Phylogenetic analysis of the complete L-protein sequences placed DTV within the genus *Tupavirus* (Figure 2). The closest BLAST (https://blast.ncbi.nlm.nih.gov/Blast.cgi) match to the assembled DTV genome was Wenzhou Myotis laniger tupavirus 1 (GenBank accession no. OM030290.1), with an overall genomewide nucleotide identity of 50.72%. Amino acid sequence divergence in the nucleocapsid protein between the DTV and the closest BLAST match (Wufeng bat tupavirus 2; accession no. OQ715690.1) was 45.12%. Amino acid sequence divergence between the DTV and the closest BLAST match was 69.38% (Wufeng bat tupavirus 2; accession no. OQ715690.1) in the G protein and 44.64% (Klamath virus; accession no. KM204999.1) in the L protein. Virus isolation with multiple passages for >1 month using 6 cell lines from diverse host species was unsuccessful, as indicated by the lack of visible cytopathic effect and negative results with a DTV PCR (Appendix 2 Table 3).

## Conclusions

We report the identification and characterization of a novel rhabdovirus from the brain of a stranded Atlantic white-sided dolphin with severe encephalitis. DTV is genetically distinct from previously known cetacean rhabdoviruses, and phylogenetic analysis indicates a considerable gap between DTV and other tupaviruses, suggesting a substantial level of unsampled viral diversity within the group. Based on the presented genomic divergence, genome organization, phylogenetic placement, and host species, DTV complies with International Committee on Taxonomy of Viruses demarcation criteria as a new species within the genus *Tupavirus* (*10*).

Reports of rhabdoviruses in aquatic mammals are scarce. Their detection and discovery are hindered by numerous challenges presented by the environment, host biology, and postmortem decomposition; therefore, even single case reports of a new virus from an aquatic mammal host provide valuable information to enhance pathogen surveillance in aquatic environments.

Unlike the previous 2 cases of cetacean rhabdovirus infection, DTV was associated with signs of severe disease. The severity of encephalitis we described was striking, but not unique. Many pathogens, including morbillivirus and *Brucella ceti* bacteria, can cause encephalitis in marine mammals (*12*). Those agents and others were ruled out in this case through a series of PCR tests, further supporting DTV as the causative pathogen. Even so, we cannot totally rule out the presence of concurrent infections. DTV isolation in cultured cells was challenging and unsuccessful; however, it is critical for experimental infections in animal models that will support investigations into DTV’s host range, tissue tropism, transmission, and pathogenicity. PCR screening and whole-genome sequencing methods for DTV could enable DTV surveillance. In addition, metagenomic sequencing of both affected and unaffected tissues might identify any co-infections.

The apparent preference of DTV for specific areas of the brain is reminiscent of rabies infection of terrestrial mammals, in which different host species exhibit unique patterns of lesion distribution in the brain; those patterns are an important criterion for disease recognition and diagnosis (*13*). A large outbreak of rabies has been reported in cape fur seals (*Arctocephalus pusillus*) in South Africa, which likely originated as a spillover event from terrestrial canids (*14*). Before that event, rabies in marine mammals was considered vanishingly rare; 1 case had been reported in a ring seal (*Pusa hispida*) in Svalbard, Norway, in 1981 (*15*).

This work highlights the importance of responding to incidents involving dead marine animals and conducting thorough investigations and diagnostics. Such discoveries can compound conservation concerns for marine species. It is imperative to continue documenting and examining cetacean stranding incidents in Canada, including those involving species not considered to be a priority by federal government wildlife managers. Our work has provided histopathologic and molecular evidence linking a cetacean rhabdovirus to CNS pathology and supports further investigation to characterize DTV and its associated pathology and epidemiology.

Appendix 1 Additional information about a novel dolphin tupavirus from a stranded Atlantic white-sided dolphin with severe encephalitis, Canada, 2024.

Appendix 2 Data from study of novel dolphin tupavirus from a stranded Atlantic white-sided dolphin with severe encephalitis, Canada, 2024.
