# Supplementary material for: Novel Dolphin Tupavirus from Stranded Atlantic White-Sided Dolphin with Severe Encephalitis, Canada, 2024
Source: Emerg Infect Dis. 2025 Nov;31(11):2144–8. doi: 10.3201/eid3111.251203 (PMC12704545; doi:10.3201/eid3111.251203)
Supplement: Appendix 1 — Additional information about a novel dolphin tupavirus from a stranded Atlantic white-sided dolphin with severe encephalitis, Canada, 2024. [file 25-1203-Techapp-s1.pdf]

Article DOI: https://doi.org/10.3201/eid3111.251203

EID cannot ensure accessibility for supplementary materials supplied by authors. Readers who have difficulty accessing supplementary content should contact the authors for assistance.

Novel Dolphin Tupavirus from Stranded Atlantic White-Sided Dolphin with Severe Encephalitis, Canada, 2024

Appendix 1

Supplementary Methods

1. *Sample Processing and Genome Sequencing*

Fresh frozen brain tissue was homogenized in 10% w/v PBS with zircon beads. Nucleic acid was extracted from the homogenate using the Quick-DNA/RNA Miniprep Plus kit (Zymo Research, Irvine, CA), including an on-column DNase I treatment. First-strand cDNA synthesis was done using the SuperScript IV First-Strand Synthesis System (Invitrogen, Waltham, MA), and second-strand synthesis used the NEBNext® Ultra II Non-Directional RNA Second Strand Synthesis Module (New England Biolabs, Ipswich, MA).

Library preparation and viral enrichment were performed using the Comprehensive Viral Research Panel (Twist Biosciences, San Francisco, CA) following the manufacturer's guidelines. The resulting library was sequenced on a NextSeq 2000 instrument using a P1 flow cell and 600-cycle reagent kit (Illumina, San Diego, CA). The 5′ and 3′ termini of the genome were sequenced by Rapid Amplification of cDNA Ends (RACE), using the SMARTer RACE 5′/3′Kit following the manufacturer's protocol (Takara Bio, Mountain View, CA). The RACE primer sequences were DTV_RACE_5′: GGCCACGATTGAAACGCAGCCTGCAT and DTV_RACE_3′: TCCACTTGAACCCGCTCCCGATCCC. The amplified 5′ and 3′ RACE products were sequenced using the Native Barcoding sequencing kit—SQK-NBD114.96 (Oxford Nanopore Technologies, Oxford, United Kingdom) and sequenced on a PromethION FlowCell—FLO-PRO114M.

2. Data Processing and Phylogenomic Analysis

2.1 *Raw Data Processing*

Raw Illumina sequence data were initially processed using an in-house, automated pipeline, nf-villumina v2.0.1 (*1*), as previously described (*2*). Raw Nanopore sequencing data were basecalled using Dorado v0.9.0 and the super-accurate base-calling model with a minimum quality score threshold of 10. The basecalled data were processed with Porechop_ABI v0.5.0 (*3*) for adaptor trimming. Finally, we used Chopper v0.5.0 (*4*) for read length filtering before consensus sequence generation using an in-house developed pipeline, nf-virontus v1.0.1 (*5*).

Assembled contigs from both Illumina and Nanopore were further processed in Geneious Prime v2025.0.3 for both iterative mapping with Geneious mapper on medium-low sensitivity with up to five iterations and sequence alignment using MAFFT v7.490 (*6*,*7*) under the default settings. The final consensus was manually edited to generate the full-length genome. A combination of ORF finder and protein BLAST was used to annotate the DTV genome. The final genome assembly statistics were calculated in Geneious Prime v2025.0.3.

2.2 *Phylogenetic Analysis*

The phylogenetic placement of the dolphin tupavirus was assessed using the complete amino acid sequences of the L protein, conventionally used in the classification and phylogenetics of rhabdoviruses (*8*). The initial broad-scale phylogenetic analysis included 438 L protein sequences across Rhabdoviridae, encompassing representative viruses from each recognized genus within the family (n = 62 genera). Subsequently, a fine-scale analysis was conducted focusing on the Tupavirus genus group, including the novel dolphin tupavirus. The Tupavirus genus analysis dataset included 17 sequence records, 14 L-protein amino acid sequences from all publicly available tupaviruses and 3 outgroup species from the genus Sunrhavirus. All sequence records for the phylogenetic analysis were downloaded from the GenBank public repository (accessed on March 23, 2025) (Appendix 2 Table 1, https://wwwnc.cdc.gov/EID/article/31/11/25-1203-App2.xlsx). Multiple sequence alignment was performed using MAFFT v.7.511 (*6*,*7*,*9*) under the default settings.

Phylogenetic analysis was performed using maximum likelihood implemented in IQTREE v2.2.3 (*10*). The best-fit model of sequence evolution was selected based on the Bayesian information criterion (BIC) score (*11*) calculated by ModelFinder (*12*).

Q.pfam+F+I+R10 and LG+F+I+G4 models were selected as the best-fitting for rhabdovirus and tupavirus datasets, respectively. Node support was estimated by ultrafast bootstrap (*13*) and the SH-aLRT test (*14*) with 1,000 replicates each.

References

1. Kruczkiewicz P, Lung O. CFIA-NCFAD/nf-villumina v2.0.1. 2022 Aug 10 [cited 2024 Jan 23]; https://zenodo.org/records/10557911.
2. Fisher M, Nebroski M, Davies J, Janzen E, Sullivan D, Lung O. Discovery and comparative genomic analysis of a novel equine anellovirus, representing the first complete *Mutorquevirus* genome. Sci Rep. 2023;13:3703. PubMed https://doi.org/10.1038/s41598-023-30875-7
3. Bonenfant Q, Noé L, Touzet H. Porechop_ABI: discovering unknown adapters in Oxford Nanopore Technology sequencing reads for downstream trimming. Bioinforma Adv. 2023 Jan 1;3:vbac085.
4. De Coster W, Rademakers R. NanoPack2: population-scale evaluation of long-read sequencing data. Bioinformatics. 2023;39:btad311. PubMed https://doi.org/10.1093/bioinformatics/btad311
5. Kruczkiewicz P. peterk87/nf-virontus. 2020 [cited 2020 Dec 22]. https://github.com/peterk87/nf-virontus.
6. Katoh K, Misawa K, Kuma K, Miyata T. MAFFT: a novel method for rapid multiple sequence alignment based on fast Fourier transform. Nucleic Acids Res. 2002;30:3059–66. PubMed https://doi.org/10.1093/nar/gkf436
7. Katoh K, Standley DM. MAFFT multiple sequence alignment software version 7: improvements in performance and usability. Mol Biol Evol. 2013;30:772–80. PubMed https://doi.org/10.1093/molbev/mst010
8. Walker PJ, Freitas-Astúa J, Bejerman N, Blasdell KR, Breyta R, Dietzgen RG, et al.; Ictv Report Consortium. ICTV virus taxonomy profile: Rhabdoviridae 2022. J Gen Virol. 2022;103:001689. PubMed https://doi.org/10.1099/jgv.0.001689
9. Katoh K, Rozewicki J, Yamada KD. MAFFT online service: multiple sequence alignment, interactive sequence choice and visualization. Brief Bioinform. 2019;20:1160–6. PubMed https://doi.org/10.1093/bib/bbx108
10. Minh BQ, Schmidt HA, Chernomor O, Schrempf D, Woodhams MD, von Haeseler A, et al. IQ-TREE 2: new models and efficient methods for phylogenetic inference in the genomic era. Mol Biol Evol. 2020;37:1530–4. PubMed https://doi.org/10.1093/molbev/msaa015

11. Schwarz G. Estimating the dimension of a model. Ann Stat. 1978;6:461–4.

12. Kalyaanamoorthy S, Minh BQ, Wong TKF, von Haeseler A, Jermiin LS. ModelFinder: fast model selection for accurate phylogenetic estimates. Nat Methods. 2017;14:587–9. PubMed https://doi.org/10.1038/nmeth.4285

13. Hoang DT, Chernomor O, von Haeseler A, Minh BQ, Vinh LS. UFBoot2: improving the ultrafast bootstrap approximation. Mol Biol Evol. 2018;35:518–22. PubMed https://doi.org/10.1093/molbev/msx281

14. Guindon S, Dufayard JF, Lefort V, Anisimova M, Hordijk W, Gascuel O. New algorithms and methods to estimate maximum-likelihood phylogenies: assessing the performance of PhyML 3.0. Syst Biol. 2010;59:307–21. PubMed https://doi.org/10.1093/sysbio/syq010

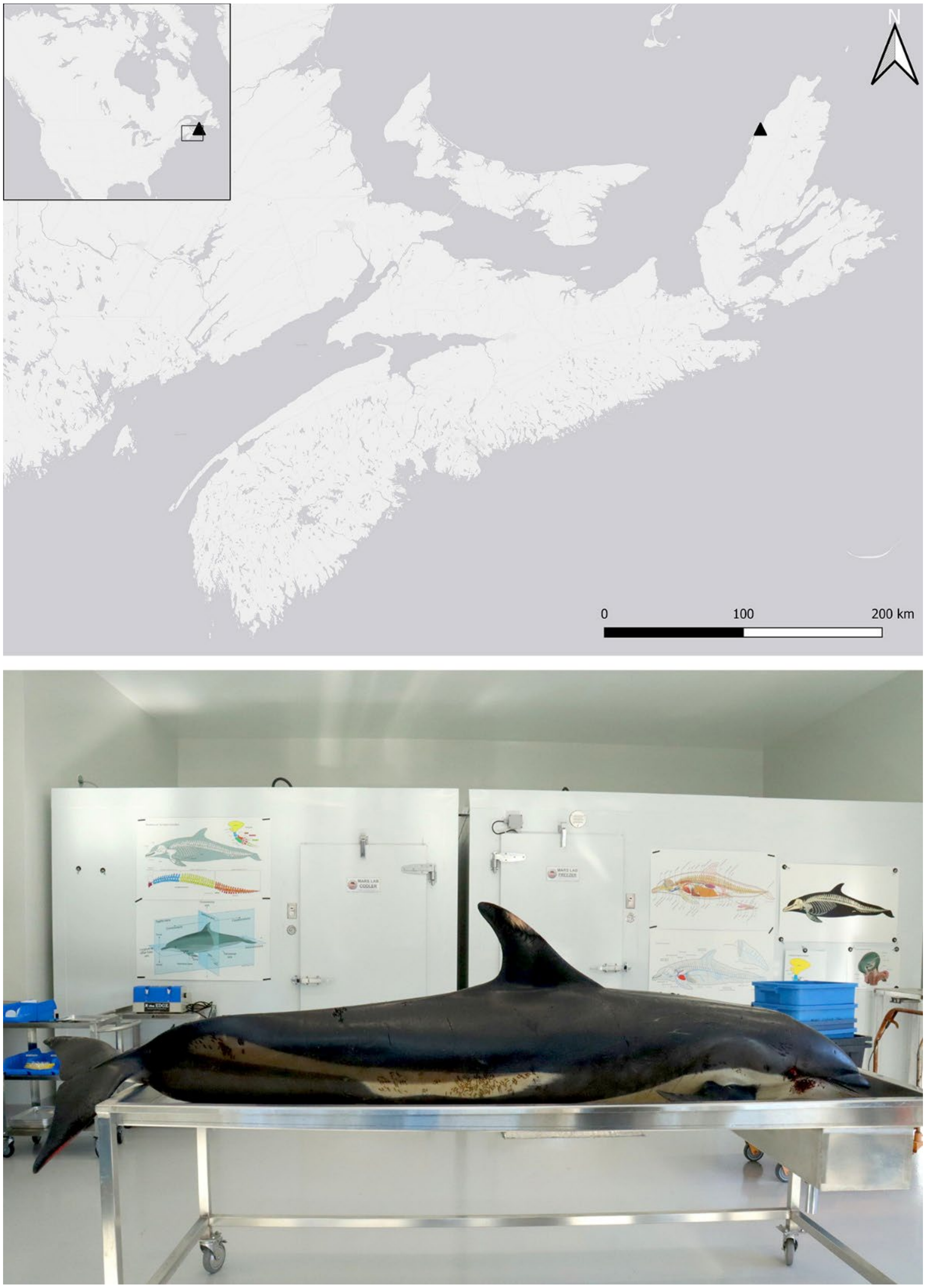

Appendix 1 Figure 1. Location map and well-preserved carcass of the collected Atlantic white-sided dolphin. A) Location of dead carcass of an Atlantic white sided dolphin at La Bloc Beach in the Cape Breton Highlands National Park, Nova Scotia, on Oct 27, 2024. B) Presented is an adult male Atlantic white sided dolphin (*L. acutus)* that was found dead on shore. The carcass was in an excellent state of preservation with minimal scavenging and postmortem decomposition (Geraci code 2).

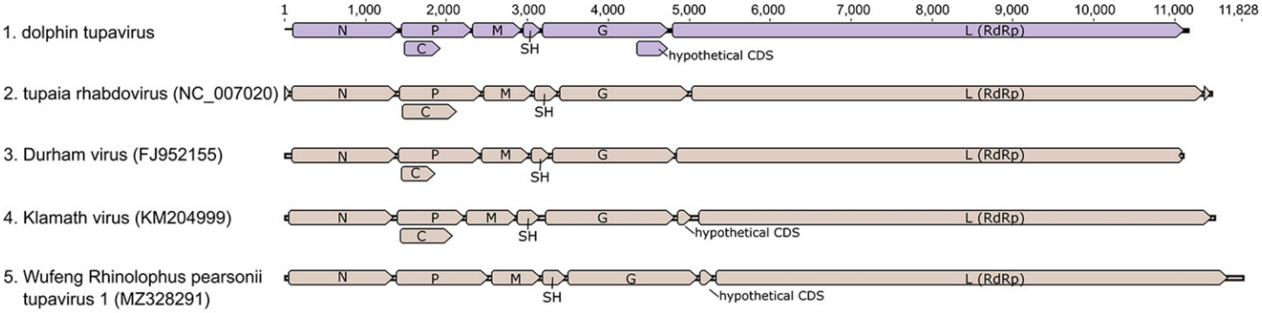

Appendix 1 Figure 2. Dolphin tupavirus genome layout and organization compared to that of other members of the *Tupavirus* genus.
